# Modeling smooth muscle cell–endothelial cell crosstalk in abdominal aortic aneurysms using 3D microvessels on-chip

**DOI:** 10.1530/VB-26-0005

**Published:** 2026-05-07

**Authors:** Philipp C Hauger, Karlijn B Rombouts, Marc Vila Cuenca, Albert van Wijk, Max C Overboom, Jan Willem Buikema, Kak Khee Yeung, Valeria V Orlova, Peter L Hordijk

**Affiliations:** ^1^ Department of Physiology, Amsterdam University Medical Centers, Amsterdam, The Netherlands; ^2^ Amsterdam Cardiovascular Sciences, Atherosclerosis and Aortic Diseases, Amsterdam, The Netherlands; ^3^ Department of Anatomy and Embryology, Leiden University Medical Center, Leiden, The Netherlands; ^4^ Department of Surgery, Amsterdam University Medical Centers, Amsterdam, Location University of Amsterdam, Amsterdam, The Netherlands; ^5^ Department of Cardiology, Amsterdam University Medical Centers, Amsterdam, The Netherlands

**Keywords:** microvasculature, stem cells, vascular disease, biotechnology, organ-on-chip

## Abstract

Abdominal aortic aneurysms (AAAs) are pathological dilations of the abdominal aorta. To date, surgical intervention has been the only option for managing large AAAs, with no pharmacological therapies to prevent growth of small aneurysms. A current limitation in investigating further pharmacological avenues is the translatability of results from either animal models or patient trials that are limited by co-morbidities and disease severity. To bridge this knowledge gap, we created a novel patient-specific vessel-on-chip (VoC) model of the microcirculation in AAA (AAA-VoC), to specifically address VSMC–EC crosstalk. We found that co-culture of both C (control)-VSMCs and AAA-patient-derived VSMCs with healthy, hiPSC-derived ECs generate lumenized and perfusable microvascular networks. We show that AAA-VoCs are characterized by an enlarged average vascular diameter. We furthermore found that AAA-VSMCs show phenotypical deviations from C-VSMCs after 7 days in co-culture such as increased number and surface area, indicative of a preserved pathological phenotype in our *in vitro* model. Finally, we demonstrate that AAA-VoCs show an elevated level of pro-inflammatory cytokine expression and an impaired endothelial barrier function, resulting in vascular leakage. With this study, we show that AAA-VSMCs affect microvascular networks formed by healthy hiPSC-ECs and that a AAA-VSMC phenotype is preserved in 3D co-culture, making this model valuable for future studies investigating treatments for AAA.

## Introduction

Abdominal aortic aneurysms (AAAs) are pathological dilations of the aorta in the abdomen. AAAs typically remain asymptomatic until rupture, which is associated with an overall mortality rate of <80% (1). AAA patients with a known risk of rupture, currently predicted based on aortic diameter, are treated with open surgery or less invasive endovascular stent grafting procedures. While larger aneurysms are treated invasively, there is currently no therapeutical option to treat small or asymptomatic aneurysms (2). Moreover, pharmacological interventions are currently under investigation, but so far have been unsuccessful in slowing, stopping, or reducing aneurysm progression (3, 4, 5).

The lack of current pharmacological advances has raised the question for translatability of results obtained in animal models and in human observational trials and genetic studies (5). A significant limitation of animal models for AAA is that unlike humans, commonly used laboratory animals do not spontaneously develop AAA due to lifestyle factors alone, necessitating artificial disease induction methods that accelerate disease onset and introduce confounding effects related to the intervention itself (6). Furthermore, human clinical trials are limited by low and medically fragile patient populations, high rate of co-morbidities, AAA growth progression and variability, and have so far failed to produce meaningful pharmaceutical outcomes (2, 7, 8, 9, 10).

The pathogenesis of AAA involves inflammation (11), extracellular matrix (ECM) remodeling (12), and hemodynamic alterations (13) that lead to a weakening of the aortic vascular wall. AAA involves immune cells, vascular smooth muscle cells (VSMCs), and endothelial cells (ECs), however, their distinct role and contribution to disease onset and progression are not well defined (14).

In addition to immune cells, also VSMCs actively contribute to cytokine production, ECM degradation, and phenotypic modulation in AAA (15, 16). The abdominal aortic wall is perfused by the vasa vasorum, a microvascular network normally confined to the adventitial layer (17). In AAA, however, the vasa vasorum penetrates into the medial layer, consisting of VSMCs (17, 18), implying the vasa vasorum in AAA pathology (17, 19, 20, 21).

Multicellular approaches have been developed to study cellular crosstalk in AAA, by seeding cells on bioengineered scaffolds or ECM-like gels (22, 23). However, these techniques rely on defined structural matrices and tissue engineering geometries, constraining cells from self-organizing and limiting their ability to recapitulate key morphogenetic processes and microenvironmental cues.

Increasing complexity in *in vitro* tissue engineering enabled the use of self-organized 3D vessel-on-a-chip (VoC) models, in which ECs and VSMCs are embedded in a hydrogel as a single-cell suspension after which they form, over the course of several days, a vascular network with a perfusable lumen (24).

In this study, we, therefore, created a novel, patient-specific AAA-VoC model that focuses on VSMC–EC crosstalk within a vasa-vasorum-like microcirculatory environment.

**Figure 1:**
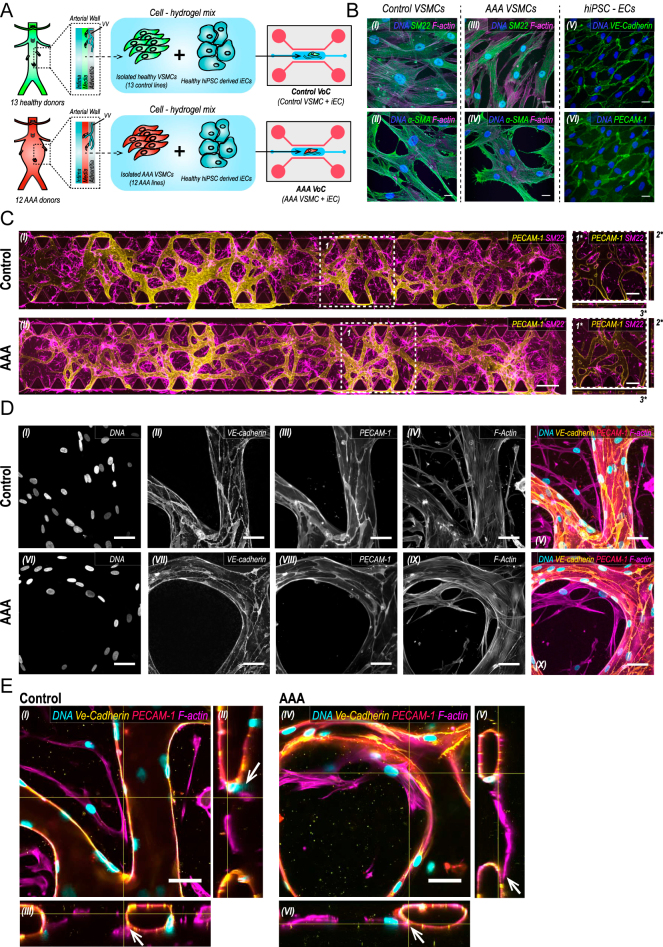
Characterization of the self-organizing VoC models. (A) Schematic overview of experimental setup. Three-layered arterial wall including intima, media, and adventitia, with contact points between VSMCs of the media and ECs of the VV (vasa vasorum). Dashed arrow indicates media as site of biopsy for primary VSMCs used in this study. Primary VSMCs, hiPSC-ECs, and hydrogel are located in the middle channel of the microfluidic unit indicated in blue; top- and bottom-flanking channels contain cell culture media, indicated in red. (B) Representative confocal images of primary VSMCs immunostained for SM22 and aSMA in control (I + II) and AAA-VSMCs (III + IV) (blue: DAPI, green: SM22 (I + III)/aSMA (I + IV), and magenta: F-actin), and hiPSC-ECs immunostained for the endothelial markers VE-cadherin and PECAM-1 (V + VI) (blue: DAPI, green: VE-cadherin (V)/PECAM-1 (IV), and magenta: F-actin). Scale bars: 20 μm. (C) Representative confocal images of microvascular networks showing hiPSC-ECs (yellow: PECAM-1) and primary VSMCs (magenta: SM22) across the complete length of the microfluidic channel. Upper panel C-VoC; lower panel AAA-VoC. Scale bars: 500 μm. Dashed square: (1*) cross-sectional images in xyz, (2*) xy, and (3*) yz from a representative area (dashed square, 1). Scale bars: 250 μm. (D) Higher-magnification confocal images of microvascular structures, upper panel in C-VoC, lower panel in AAA-VoC. (I + VI) Cell nuclei, (II + VII) VE-cadherin, (III + VIII) PECAM-1, and (IV + IX) F-actin. (V) Composite images for C-VoC and (X) AAA-VoC (blue: DAPI, yellow: VE-cadherin, red: PECAM-1, and magenta: F-actin). Scale bars: 50 μm. (E) Representative confocal orthogonal images of (I) C-VoC in xyz and (IV) AAA-VoC in xyz depicting contact points between primary VSMCs and hiPSCs in VoC culture (contact points indicated by white arrows in xy and yz slices, C-VoC: II + III, AAA-VoC: V + VI) (blue: DAPI, yellow: VE-cadherin, red: PECAM-1, and magenta: F-actin). Scale bars: 50 μm.

By co-culturing primary VSMCs from either AAA patients (AAA-VSMC) or healthy individuals (C-VSMC), with healthy human induced pluripotent stem cell-derived ECs (hiPSC-ECs), we established a controlled platform to investigate how patient-derived VSMCs influence microvascular network formation and EC behavior (Fig. 1A). While this model does not reproduce the full cellular complexity of AAA, it does allow focused investigation of VSMC–EC crosstalk. We show that AAA-VSMCs alter microvascular morphology and hiPSC-EC function, including altered VSMC proliferation and a decrease in EC junctional integrity, indicating that aspects of the AAA-VSMC phenotype remain preserved in 3D co-culture. This AAA-VoC model provides a valuable tool to dissect VSMC–EC interactions and explore novel therapeutic approaches targeting VSMC and EC dysfunction in AAA.

## Methods

### Patient population, aortic tissue collection, and primary VSMC isolation

Biopsies at the largest diameter of the aorta were obtained from 12 AAA patients during open aneurysm repair in the Amsterdam University Medical Center, the Netherlands. All patients were over 18 years of age and signed informed consent (patient characteristics: Table S1 (see section on Supplementary materials given at the end of the article)). Control aortic biopsies were obtained from non-dilated infrarenal aorta of 13 post-mortem kidney donors. Biopsies were transported directly after collection on ice-cold sterile 0.9% NaCl solution from the operating room to the laboratory. Tissue collection was in accordance with the regulations of the WMA Declaration of Helsinki and Amsterdam UMC (Biobank 2017.121: Aortic Aneurysms, Atherosclerosis, and Biomarkers). As the kidney donors remained anonymous, only age and sex were reported for the control group. Patient characteristics for the two groups are as follows: i) control: 54.5 ± 15.8 years, 30.3% male, renal dysfunction: N/A and ii) AAA: 68.6 ± 10.3 years, 71.4% male, 28.57% renal dysfunction. A list of included VSMCs used in each experiment is shown in Tables S1 and S2. VSMCs were isolated from aortic biopsies and cultured *in vitro* as described previously (25). VSMCs were used between passages 1 and 8 in all experiments.

### hiPSC line culture and maintenance

The hiPSC line SCVI-111 used in this study stems from the Stanford Cardiovascular Institute (SCVI) Biobank. SCVI111 was Sendai virus reprogrammed from 490 peripheral blood mononuclear cells; the donor was a healthy male with a normal karyotype. hiPSCs were maintained on Vitronectin (StemCell Technologies, Canada)-coated suspension plates (Greiner, Austria) in mTeSR Plus (StemCell Technologies). Cells were passaged as colonies using Gentle Cell Dissociation Reagent according to the manufacturer’s instruction (StemCell Technologies); media change was performed daily.

### Differentiation of hiPSC-ECs

hiPSCs were directed toward an EC lineage following previously established protocols (26, 27, 28) with minor modifications. For mesoderm induction (day 0–3), mTeSR plus was replaced with B(P)EL medium containing 8 μM CHIR99021 (Tocris Bioscience, UK). From day 3, vascular specification was induced using B(P)EL supplemented with VEGF (50 ng/mL, Peprotech, USA) and SB431542 (10 μM, Tocris Bioscience), with medium changes on days 3, 6, and 9. On day 10, ECs were isolated via CD31-Dynabeads (Thermo Fisher Scientific, USA), as described (26, 27). Purified hiPSC-ECs were expanded in EC-SFM (Gibco, USA) supplemented with 1% human platelet-poor serum (Sigma, USA), VEGF (30 ng/mL), and bFGF (20 ng/mL, Miltenyi, Germany), and cryopreserved at passage 1 in 40% ECGM-2 (Promocell, Germany), 50% FBS (Gibco), and 10% DMSO (Sigma).

### Immunocytochemistry

Cells cultured in 2D (ibidi μ-slide 8 well, 0.1% gelatin-coated) or as microvascular networks in microfluidic chips were fixed with 4% paraformaldehyde at room temperature (15–30 min), followed by PBS washes. Samples were permeabilized with Triton X-100 (0.2–0.5%) and blocked in PBS containing 1–2% serum albumin (HSA or BSA). Primary antibodies (Table S3) were applied for 1 h at room temperature or overnight at 4°C, followed by PBS washes and incubation with appropriate secondary antibodies for 1–2 h at room temperature. Imaging was performed using a Nikon AXR confocal microscope. Image processing and 3D reconstruction were conducted using Imaris (v10.2.0) or NIS-Elements (v5.42.04).

### RNA isolation, reverse transcription, and quantitative PCR

Total RNA from ECs and VSMCs was isolated using the Direct-zol RNA MiniPrep kit. RNA quality was assessed by NanoDrop, and cDNA was synthesized using the iScript kit. qPCR was performed on a CFX Maestro system (v5.0) in technical duplicates across three plates per cell line. Gene expression was calculated using the ΔCq method (Cq_target – Cq_GAPDH), with fold change determined as 2^−ΔCq. Disease samples were normalized to the mean control fold change. Primer sequences are listed in Table S4.

### Setting up microvascular models on chip

C-VoCs and AAA-VoCs were generated as previously described with minor adjustments (24). Briefly, commercially available microfluidic devices (IdentX9, AIM Biotech, Singapore) were employed. For microvascular network formation, primary C-VSMCs or AAA-VSMCs were combined with hiPSC-ECs to achieve a cell master mix suspension containing 10 × 10^6^ hiPSC-ECs/mL and 2 × 10^6^ VSMCs/mL, resulting in a 5:1 EC-to-VSMC ratio. The cell suspension master mix was prepared in ECGM-2 medium supplemented with VEGF (50 ng/mL) and thrombin (4 U/mL, Sigma-Aldrich, USA). Prior to loading, one part of a cell suspension master mix was combined with an equal volume of ECGM-2 containing VEGF (50 ng/mL) and thrombin (4 U/mL), followed by a 1:1 dilution with fibrinogen (Sigma-Aldrich) to reach a final fibrinogen concentration of 3 mg/mL. Immediately after, 15 μL of the resulting cell-hydrogel mixture were added into the central channel of the IdentX9 chip and allowed to polymerize for 15 min at RT. To establish gravity-driven perfusion, 100 μL of VEGF-supplemented ECGM-2 were added to the right media inlet and 50 μL to the left. The medium was refreshed daily using the same approach, maintaining gravity-driven flow for 7 days. On the first day of culture, the medium was supplemented with the γ-secretase inhibitor DAPT (10 μM, Tocris Bioscience).

### Permeability assessment of microvascular networks

Endothelial barrier function was assessed in VoCs using a FITC–dextran permeability assay. Vascular networks were labeled with UEA-I (1:600, 1 h, 37°C) and imaged live on a Nikon AXR confocal microscope under environmental control. FITC–dextran (70 kDa, 2 μg/mL) was added to the device inlets, and fluorescence images were acquired every 10 min for 50 min. Permeability was quantified in NIS-elements by measuring FITC–dextran mean fluorescence intensity in UEA-I-positive vessels and surrounding ECM regions, and calculating a leakage index (vessel/ECM fluorescence ratio) over time.

### Geometry analysis of microvascular networks

On day 7 of culture, vascular networks were fixed and subjected to immunostaining using either UEA-I or PECAM1 to label endothelial structures. Overview images of microvascular networks were generated and subsequently transformed to as a maximum intensity projection in Z. Image segmentation was carried out in CellProfiler software (version 4.2.1, Broad Institute). The resulting binary images were analyzed in ImageJ (NIH, USA) using the open-source DiameterJ plugin (29). All image processing and quantitative analyses followed identical workflows to ensure consistency across experimental groups.

### Shear stress application on hiPSC-ECs in 2D

A computer-controlled pump system (ibidi), consisting of a pump, fluidic unit, and perfusion set (15 cm tubing, 1.6 mm inner diameter, 10 mL reservoirs), was employed to culture hiPSC-ECs under laminar flow. Cells were seeded into fibronectin (Merck, Germany) coated μ-slides VI 0.6 (ibidi) at a density of 0.5 × 10^6^ cells/mL. After a 24 h attachment period, the slides were connected to the perfusion system, and laminar flow was applied gradually, starting from 1 h at 2.5 dyn/cm^2^, then 1 h at 7.5 dyn/cm^2^, and subsequently maintained at 18 dyn/cm^2^ for 3 days. All flow experiments were performed in a standard cell culture incubator (37°C, 5% CO_2_), in EC-SFM supplemented with 1% human platelet-poor serum, VEGF (30 ng/mL), and bFGF (20 ng/mL).

### Determination of polarity index (PI)

EC alignment was evaluated by quantifying Golgi–nucleus orientation to calculate a polarity index (PI; mean resultant length of angles). Golgi, cell borders, and nuclei were stained for Golgin-97, VE-cadherin, and DAPI, respectively. Angles were measured in ImageJ by drawing vectors from the nucleus center to the Golgi, referenced to a defined axis (parallel to image borders in 2D cultures or vessel walls in microvascular networks). For VoCs, vessels were additionally categorized into straight and branching regions prior to analysis (Fig. S1).

### Calcium transient determination

Two hours prior to the assay, culture medium was replaced with a mixture consisting of i) ECGM-2 medium supplemented with VEGF (50 ng/mL) and UEA-I, and ii) 5 μM Cal-520 (Abcam, UK) in 0.02% Pluronic F-127 (Sigma-Aldrich). Live-cell imaging was conducted using a Nikon AXR confocal microscope equipped with environmental control (37°C, 5% CO_2_). Baseline calcium activity was recorded for 2 min at 3-s intervals. Endothelin-1 (ET-1) was then introduced into the media inlets of the microfluidic platform to achieve a final concentration of 10 nM. After a 5-min incubation period, the same imaging coordinates were revisited, and calcium dynamics were recorded for an additional 2 min at the same acquisition interval. ROIs corresponding to VSMCs were identified based on peak calcium loading following ET-1 stimulation (Fig. S2). Quantification was performed on z-stack images, and the MFI of Cal-520 was measured at each time point for individual ROIs to assess temporal calcium fluctuations.

### Cytokine detection array

Proteome Profiler Human Cytokine Array Kit (R&D Systems, USA) was used to quantify the cytokine levels in VoC supernatants. Supernatants were collected from three VoCs (control, AAA) 24 h after the final media exchange and pooled before processing. The arrays were performed following the instructions provided by the manufacturer with one modification where SuperSignal™ West Femto Maximum Sensitivity Substrate from Thermo Fisher Scientific was applied for the detection and imaging of the resulting dot blot signals.

### Statistical analysis

All functional assays on VoCs were performed on culture day 7. Statistical analyses were performed using GraphPad Prism version 10 (GraphPad Software, USA). Data are presented as mean ± SEM or mean ± SD. Data were tested for normality, and appropriate parametric or non-parametric tests were applied accordingly. For longitudinal data, repeated-measures analyses were used where appropriate. Statistical significance was set at *P* < 0.05 (*P < 0.05, **P < 0.01, ***P < 0.005, ****P < 0.001).

## Results

### C-VSMCs and AAA-VSMCs support hiPSC-EC vascular network formation in 3D

In this study, we generated a 3D *in vitro* co-culture model of the microvasculature in the AAA aortic wall using a healthy donor-derived hiPSC-EC line in co-culture with primary C-VSMCs or primary AAA-VSMCs (Fig. 1A). In 2D *in vitro* culture, C-VSMCs and AAA-VSMCs express canonical VSMC markers, including smooth muscle protein 22-alpha (SM22α) and alpha-smooth muscle actin (αSMA) (Fig. 1B). hiPSC-ECs form a confluent monolayer and express key endothelial markers, including vascular endothelial cadherin (VE-cadherin) and platelet endothelial cell adhesion molecule-1 (PECAM-1/CD31) (Fig. 1B).

We found that co-culture of hiPSC-ECs with C-VSMCs and AAA-VSMCs resulted in vascular networks after 7 days (Fig. 1C). For both C-VoCs and AAA-VoCs, we observed vascularization of the entire hydrogel chamber (Fig. 1C). We furthermore observed that both C-VSMCs and AAA-VSMCs enabled vascular lumen formation (Fig. 1C). VSMCs remained viable for 7 days of co-culture and retained their characteristic phenotype as indicated by SM22 staining. Microvessels showed organized adherens (AJ) and tight (TJ) junctions in both C-VoCs and AAA-VoCs structures, identified by VE-cadherin, PECAM-1, and Claudin-5 expression (Figs 1D, S3). Moreover, we assessed whether hiPSC-ECs and VSMCs establish heterotypic cell–cell contact using generic and EC-specific immunostaining. After 7 days of co-culture, we observed multiple contact points between VSMCs and hiPSC- ECs (Fig. 1E), throughout the entire hydrogel channel and for both C-VoCs and AAA-VoCs.

### AAA-VoCs show enlarged vascular diameter

**Figure 2:**
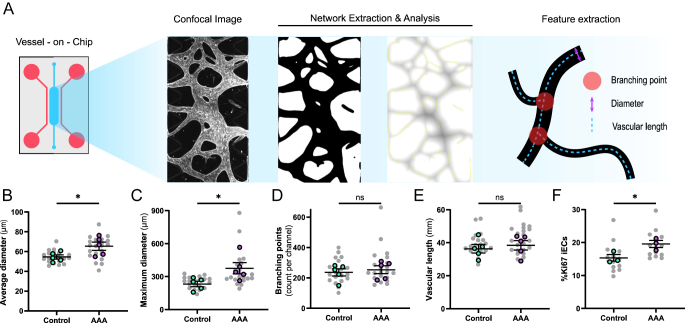
Geometry of microvasculature in VoC models. (A) Schematic overview of workflow for geometry analysis in VoC models. (B, C, D, E) Quantification of microvascular geometry in VoC models. (B) microvascular diameter in μm, (C) maximal vascular diameter in μm, (D) number of branching points per microfluidic channel, and (E) total vascular length in μm. Data are shown as mean ± SEM. Colored data points represent five independent experiments on five (control, green) and four (AAA, magenta) independent VSMC donors; data points in gray represent technical replicates, 2–5 VoC channels per experiment were imaged and data were averaged to yield one value per donor. Statistics analysis was performed on the donor-averaged values. Data passed Shapiro–Wilk normality test, and groups were compared using an unpaired two-tailed *t*-test. *P < 0.05. (F) Quantification of a fraction of Ki67-positive hiPSC-ECs nuclei at day 7 in %. Data are shown as mean ± SEM. Data points represent 3 independent experiments on VoCs with 3 (control) and 3 (AAA) independent VSMC lines; 3–6 VoC channels per experiment were imaged and data averaged to yield one value per cell line. Data passed Shapiro–Wilk normality test, and groups were compared using an unpaired two-tailed *t*-test. *P < 0.05.

We next aimed at characterizing the geometry of the microvascular structures generated by hiPSC-ECs in co-culture with either C-VSMCs or AAA-VMCs (Fig. 2A). Morphometric analysis showed that the hiPSC-ECs in co-culture with AAA-VSMCs generated microvascular structures with increased average vessel diameters (Fig. 2B). We furthermore found that the maximum vessel diameter was also increased in AAA-VoCs compared to C-VoCs (Fig. 2C). Moreover, we quantified branching points and vessel length but did not find significant differences (Fig. 2D and E). We furthermore investigated whether the increased vessel diameter in AAA-VoCs correlates with increased hiPSC-EC proliferation.

We stained VoC-cultures for Ki67, and identified hiPSC-ECs using UAE-1 (Fig. S4). We found a significantly increased fraction of Ki67-positive hiPSC-EC nuclei in AAA-VoCs over C-VoCs (Fig. 2F). Overall, these results indicate that both C-VSMCs and AAA-VSMCs support vascular network formation of comparable complexity, but that the vascular diameter is increased when hiPSC-ECs are co-cultured with AAA-VSMCs, in line with increased hiPSC-EC proliferation.

### hiPSC-ECs align with gravity-driven flow in both C-VoCs and AAA-VoCs

A hallmark of viable ECs is their alignment under atheroprotective laminar shear stress (LSS), and the reduction of the alignment in areas of turbulent flow (30). However, flow alignment is also linked to crosstalk with the microenvironment of the aortic wall, primarily composed of VSMCs (31, 32). Therefore, a lack of hiPSC-EC polarization under flow may reflect not only impaired mechanoresponsiveness but also a dysregulated VSMC phenotype.

**Figure 3:**
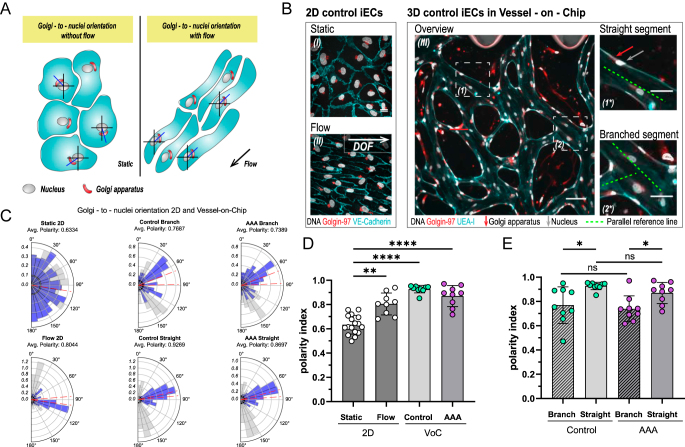
Endothelial polarity in the VoC models. Flow response of hiPSC-ECs. In 2D, cells were exposed to 18 dynes/cm^2^ laminar shear stress, in 3D VoCs gravity-driven flow was established by daily application of a 100–50 μL liquid gradient. (A) Schematic overview of EC Golgi-to-nuclei orientation in static conditions (left) and upon laminar shear stress exposure (right). Golgi-to-nuclei angles (blue line) indicate the angle used to calculate the polarity index (PI). (B) Representative confocal images of hiPSC-ECs in 2D for Golgi-to-nuclei orientation analysis (left panel, (I) static, (II) flow), (gray: DAPI, red: Golgin-97, and cyan: VE-cadherin). Scale bars: 20 μm. DOF = direction of flow. (III) Representative confocal image of VoC for Golgi-to-nuclei orientation analysis. Scale bars: 100 μm. (1*) Images of a representative straight labeled segment and (2*) branched segment derived from indicated areas (1) and (2), respectively. Green dashed lines: reference lines for Golgi-to-nuclei angle determination, red arrow: Golgi, and gray arrow: nuclei (gray: DAPI, red: Golgin-97, and cyan: VE-cadherin). Scale bars: 50 μm. (C) Radial histograms depicting the distribution of Golgi-to-nuclei orientation in hiPSC-ECs for static and flow 2D (first column), C-VoC branched and straight areas of the microvasculature (middle column) and AAA-VoC branched and straight areas of the microvasculature (right column). Blue: transformed angles, gray: original angles, and red dashed lines: lower and upper limits of the 95% confidence interval of the transformed orientation angles. Radial axis indicates the density (frequency) of observations normalized to unit area. (D and E) Quantification of Golgi-to-nuclei orientation. (D) PI comparison in 2D static and flow, and straight branches of C-VoC and AAA-VoC, (E) PI in branched and straight areas of C-VoC and AAA-VoC. (D) Data are shown as mean ± SD. Data points in the graph represent for Static 2D: 3 independent experiments (5–6 fields of view); for Flow 2D: 3 independent experiments (3 fields of view each); for C- and AAA-VoC: 3 independent experiments with 3 independent VSMC lines (3 fields of view each). (E) Data are shown as mean ± SD. Data points in the graph represent 3 experiments with 3 independent VSMC lines (3 fields of view each). C-VoC (straight) failed normality (Shapiro–Wilk test, *W* = 0.7673, *P* = 0.0086). Pairwise comparisons performed using unpaired two-tailed *t*-tests for normally distributed groups and Mann–Whitney tests for non-normal groups; ****P < 0.001, ***P < 0.005, **P < 0.01, *P < 0.05.

It is known that LSS stimulates ECs to polarize, with the Golgi aligning upstream of the nucleus (Fig. 3A) (33). The polarity index (PI) was defined as the combined degree of the angles transformed to center around the vertical axis. Values close to 1 indicate strong alignment, while values near 0 reflect random orientations. We added two control groups: hiPSC-ECs in a 2D *in vitro* static culture (without flow) (Fig. 3B) and hiPSC-ECs in a 2D *in vitro* setup under defined LSS (72 h, 18 dyn/cm^2^). In VoC cultures, we furthermore separated straight vascular areas from branching points (Fig. 3B). The direction of flow (DOF) in VoCs was determined by estimating a parallel line along the vascular wall (Fig. 3B).

We found that, under static conditions, the PI of hiPSC-EC is comparably low and that the application of flow in 2D for 72 h significantly increases the PI (Fig. 3C and D), showing that these hiPSC-ECs are flow-responsive. Interestingly, we found that for straight branches in both C-VoCs and AAA-VoCs, the PI was further increased when compared to the 2D flow condition (Fig. 3D). We furthermore found a significant increase in PI in straight areas in C-VoCs and AAA-VoCs (Fig. 3E).

These findings show that both C-VSMCs and AAA-VSMCs support hiPSC-ECs in a microvascular model and that hiPSC-ECs are viable and align to flow.

### AAA-VSMCs show increased cell number and surface area at day 7 of AAA-VoC culture

**Figure 4:**
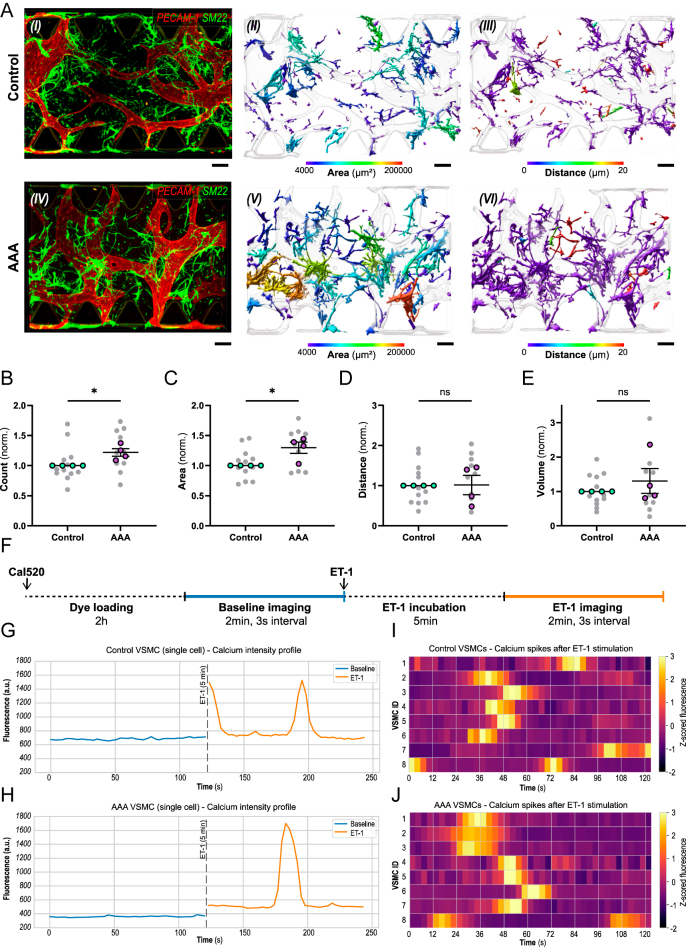
AAA-VoC VSMCs show increased cell number but no increase in distance to ECs or Ca^2+^ responsiveness. (A) Representative confocal images of (I) C-VoC and (IV) AAA-VoC showing hiPSC-ECs (red: PECAM-1) and C-VSMCs (green: SM22). Surface-rendered objects based on PECAM-1 (microvascular network, transparent render) and SM22 (VSMCs, color-coded render). (II + V) VSMCs with a color-coded scale for 3D surface area in μm^2^ and (III + VI) color-coded scale for VSMC distance to microvascular network in μm. (B, C, D, E) Quantification of VSMC characteristics in C-VoC and AAA-VoC showing (B) normalized cell count, (C) normalized surface area, (D) normalized VSMC distance to microvascular network, and (E) normalized overlapping volume between VSMCs and microvascular network. Data are shown as mean ± SEM. Colored data points represent *n* = 4 independent VSMC lines (control and AAA), each assayed in 3 separate experiments; gray data points represent 3–6 VoC channels imaged per experiment, data averaged to yield one value per cell line. AAA-VoC data points were normalized to the corresponding C-VoC processed in parallel. Data passed Shapiro–Wilk normality test. Unpaired two-tailed *t*-test. *P = <0.05. (F) Schematic overview of experimental timeline for calcium transient recordings. (G) Representative graph for ET-1 responsive C-VSMC. (H) Representative graph for ET-1 responsive AAA-VSMCs. Blue line indicates calcium activity at baseline before ET-1 stimulation, and orange line indicates calcium activity after ET-1 stimulation. Dashed line indicates incubation step of 5 min after ET-1 stimulation. (I) Heatmap with a *z*-score normalized color-code for calcium activity depicting 8 representative C-VSMCs (VSMC IDs 1–8), 5 min after ET-1 stimulation. (J) Heatmap with a *z*-score normalized color-code for calcium activity depicting 8 representative AAA-VSMCs (VSMC IDs 1–8), 5 min after ET-1 stimulation.

We then examined whether AAA-VSMCs recapitulate AAA-specific features. We employed immunostaining (PECAM-1 for ECs, SM22 for VSMCs), combined with 3D rendering and quantitative analysis (Fig. 4A). A hallmark of AAA is a VSMC phenotypic switch from a quiescent, contractile phenotype toward a proliferative, synthetic phenotype (34), and previous work identified that proliferative genes were increased in human AAA tissue (14). Interestingly, we found an increased number of AAA-VSMCs after 7 days in VoC culture compared to C-VSMCs (Fig. 4B). However, Ki67 staining showed no difference in proliferation between C-VSMCs and AAA-VSMCs at day 7 of co-culture (Fig. S5). Phenotypic switching from spindle-shaped contractile cells toward irregular synthetic, fibroblast- or macrophage-like morphologies in AAA-VSMCs suggests a change in cell morphology. We found that AAA-VSMCs show an increased surface area compared to C-VSMCs after 7 days of hiPSC-EC co-culture, supporting that hypothesis and suggestive of a preserved disease phenotype of AAA-VSMCs (Fig. 4C). We furthermore investigated whether AAA-VSMCs are aberrant in terms of distance to the microvascular network (Fig. 4D) or amount of area covered on the microvascular network (Fig. 4E), but did not find significant differences. Together, we find that AAA-VSMCs show increased numbers and a distinct morphology after 7 days in culture, while their behavior in relation to the microvascular network is comparable to C-VSMCs.

### C-VSMCs and AAA-VSMCs respond to ET-1 after 7 days of co-culture on-chip

We next investigated whether C-VSMCs and AAA-VSMCs exhibit calcium transients in response to a contractile stimulus (35), using Cal-520 AM (36, 37). We stimulated cells with ET-1 (Fig. 4F), a vasoconstrictive peptide which triggers calcium signaling in VSMCs *in vivo* (38). Fields of view were selected based on UEA-I staining, indicating vascularized hydrogel areas, and ROIs were selected for calcium-dye positive, UEA-I negative cells, representing VSMCs (Fig. S2). We then plotted the MFI for single ROIs before and 5 min after ET-1 stimulation and found ET-1-responsive cells in both C-VoCs (Fig. 4G) and AAA-VoCs (Fig. 4H). Furthermore, we found comparable calcium transient frequency and duration, indicating that both C-VoCs and AAA-VoCs contain viable and ET-1-responsive VSMCs (Fig. 4I and J). Together, these results indicate that although AAA-VSMCs have a distinct phenotype in VoCs, viability, and functionality as deduced from calcium transients remain comparable with C-VSMCs.

### AAA-VSMC supernatant induces a pro-inflammatory phenotype and variable cytokine expression in hiPSC-ECs

**Figure 5:**
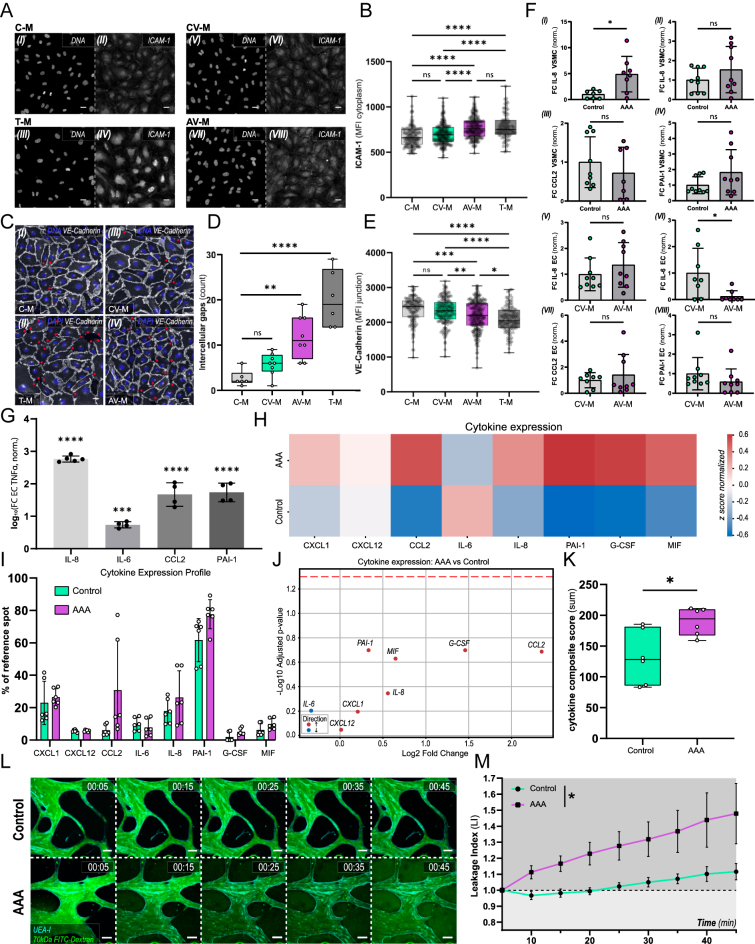
Cytokine expression and endothelial barrier in VoC models. (A) Representative confocal images of hiPSC-ECs following 5 h exposure to (I–II) fresh VSMC media (C-M), (III–IV) fresh VSMC media supplemented with 10 ng/mL TNFα (T-M), (V–VI) C-VSMC-conditioned media (72 h; CV-M), or (VII–VIII) AAA-VSMC-conditioned media (72 h; AV-M). For each pair, the left panel shows DAPI and the right panel shows ICAM-1. Scale bars: 25 μm. (B) ICAM-1 intensity quantification as cytoplasmic mean fluorescence intensity (MFI) per cell. Data are presented as boxplots with overlaid single-cell values; whiskers indicate the minimum and maximum values. All groups failed normality by Shapiro–Wilk test (all *W* < 0.98 and all *P* < 0.04). Group differences assessed using the Kruskal–Wallis test for non-parametric data; ****P < 0.0001. (C) Representative confocal images of hiPSC-ECs following 5 h exposure to (I) fresh VSMC media (C-M), (II) fresh VSMC media supplemented with 10 ng/mL TNFα (T-M), (III) C-VSMC-conditioned media (72 h; CV-M), or (IV) AAA-VSMC-conditioned media (72 h; AV-M). (C) (I–IV): DAPI (blue) and VE-cadherin (gray) with red stars indicating sites of intercellular gaps. Scale bars: 25 μm. (D) Quantification of VE-cadherin-negative, intercellular gaps is shown as the number of identified gaps per field of view. Data passed Shapiro–Wilk normality test. Group differences tested by one-way ANOVA; ****P < 0.001, **P < 0.01, ns – *P* > 0.05. (E) VE-cadherin intensity quantification as mean junctional fluorescence intensity (MFI) per cell. Data are presented as boxplots with overlaid single-cell values; whiskers indicate the minimum and maximum values. Normality was rejected for two groups by the Shapiro–Wilk test (all *W* < 0.99; *P* < 0.004 for non-normal groups). Group differences assessed using the Kruskal–Wallis test for non-parametric data; ****P < 0.001, ***P < 0.005, **P < 0.01, *P < 0.05, ns – *P* > 0.05. (F) Quantification of gene expression with qPCR of hiPSC-ECs after exposure for 18 h to CV-M or AV-M. (I) *IL-8* expression in C-VSMCs and AAA-VSMCs, (II) *IL-6* expression in C-VSMCs and AAA-VSMCs, (III) *CCL2* expression in C-VSMCs and AAA-VSMCs, (IV) *PAI-1* expression in C-VSMCs and AAA-VSMCs, (V) *IL-8* expression in hiPSC-ECs after exposure to CV-M or AV-M, (VI) *IL-6* expression in hiPSC-ECs after exposure to CV-M or AV-M, (VII) *CCL2* expression in hiPSC-ECs after exposure to CV-M or AV-M, and (VIII) *PAI-1* expression in hiPSC-ECs after exposure to CV-M or AV-M. Data are shown as fold change (2^−^ΔCq) normalized to CV-M control (control = 1) ± SD. Data points in the graph represent 3 experiments with 3 independent VSMC lines (I–IV) or 3 experiments with hiPSC-ECs exposed to conditioned media from 3 independent VSMC lines (V–VIII). Normally distributed groups were compared using Welch’s *t*-test, and non-normal groups were compared using the Mann–Whitney test; *P < 0.05, ns – *P* > 0.05. (G) Quantification of gene expression in hiPSC-ECs after 18 h exposure to T-M. Data are shown as log_10_-transformed fold change (2^−^ΔCq), normalized to C-M (control = 1), and presented as mean ± SD. Data points represent individual technical replicates (5 for *IL-8*; 4 for *IL-6*, *CCL2*, and *PAI-1*). Data passed Shapiro–Wilk normality test, with group comparison using one-way ANOVA; ****P < 0.001, ***P < 0.005, **P < 0.01, *P < 0.05. (H) The *z*-score normalized heatmap for cytokine expression levels in C-VoC and AAA-VoC supernatants, data from MFI quantification of cytokine array kit. (I) Expression levels of cytokines after MFI detection in cytokine array kit are shown as % MFI of the reference spot. Data are shown as mean ± SD, from *n* = 3 independent VSMC lines (control and AAA), each assayed in 3 separate experiments, 2 technical replicates per experiment. (J) Volcano plot showing log_2_ fold change versus –log_10_ adjusted *P*-value for cytokine expression in AAA-VoC against C-VoC, with red and blue dots indicating up- or downregulated cytokines in AAA-VoC, and the red dashed line marking the significance threshold (adjusted *P* = 0.05). (K) Composite score of cytokine expression, calculated as the sum of expression levels for all measured cytokines, compared between groups using an unpaired two-tailed *t*-test after confirming normality. *P < 0.05. Data are shown as box plots: median (line), interquartile range (box), and minimum to maximum values. Each point represents an individual sample (*n* = 3 independent VSMC lines, 2 technical replicates each). (L) Representative images of microvasculature (cyan: UEA-I) perfused with 70 kDa FITC–dextran (green) over the time course of 45 min. Top panel C-VoC, bottom panel AAA-VoC (time stamp: HH:MM). Scale bars: 100 μm. (M) Quantification of FITC–dextran leakage across the microvascular barrier in C-VoCs and AAA-VoCs, expressed as the ratio of mean MFI in the ECM to MFI in the vasculature over time. Data are shown as mean ± SEM, from 4 (control) and 5 (AAA) independent experiments; 2–6 VoC channels imaged per experiment. Tested for main effect of group across all timepoints, two-way repeated measures ANOVA. *P < 0.05.

We investigated whether VSMCs from AAA patients functionally affect healthy donor hiPSC-ECs, focusing on endothelial activation and inflammatory signaling. Four media conditions were generated: control medium (C-M), control VSMC-conditioned medium (CV-M; 72 h conditioning, *n* = 3 donors per group), AAA-VSMC-conditioned medium (AV-M; 72 h conditioning, *n* = 3 donors per group), and TNFα-supplemented medium (T-M, 10 ng/mL). hiPSC-ECs were exposed for 5 h and immunostained to visualize ICAM-1 (Fig. 5A). Analysis of the latter revealed that AV-M increased ICAM-1 levels, comparable to T-M, while C-M and CV-M showed similar low expression (Fig. 5B).

Junctional integrity was assessed by quantifying VE-cadherin-negative intercellular gaps (Fig. 5C and D) and junctional VE-cadherin mean fluorescence intensity (MFI) (Fig. 5E). AV-M increased gap formation and reduced VE-cadherin MFI compared to CV-M. T-M induced a stronger gap increase, while C-M and CV-M showed comparable effects.

Gene expression of *IL-8*, *IL-6*, *CCL2*, and *PAI-1* was analyzed in VSMCs and in hiPSC-ECs after 18 h exposure to conditioned media (Fig. 5F). In AAA-VSMCs, *IL-8* was significantly upregulated, whereas *IL-6*, *CCL2*, and *PAI-1* were comparably expressed to C-VSMCs. In hiPSC-ECs, AV-M significantly reduced *IL-6* expression without affecting *IL-8*, *CCL2*, or *PAI-1*. T-M significantly increased all four cytokines in hiPSC-ECs (Fig. 5G).

Overall, AAA-VSMC-conditioned media induced a moderate pro-inflammatory phenotype in hiPSC-ECs compared to the control VSMC media.

### Pro-inflammatory cytokines show a trend toward increased levels in 3D AAA-VoCs

To assess whether direct co-culture of hiPSC-ECs and VSMCs within our VoC model provides more definitive results, we performed a human cytokine array assay on VoC media supernatant that was collected at day 7, following 24 h of incubation on VoCs (Fig. S6). For AAA-VoC supernatant, we observed elevated levels of CXCL1, CXCL12, CCL2, IL-8, PAI-1, G-CSF, and MIF compared to C-VoC supernatant (Fig. 5H and I). To further dissect the extent of cytokine elevation in our system, we plotted a volcano plot for detected cytokines (Fig. 5J), revealing that although all cytokines (except IL-6) showed elevated levels in AAA-VoC; they are not significantly increased when observed individually.

We next asked whether the combined cytokine response might uncover a grouped difference in cytokine expression that is not apparent on a single cytokine level. To this end, we calculated a composite score by summing the concentration of all measured cytokines for either C-VoCs or AAA-VoCs, reflecting total cytokine burden. This composite score was significantly higher in AAA-VoCs compared to C-VoCs (Fig. 5K). Because cytokines differ in absolute scales, we also derived an alternative composite score by first log-transforming and then *z*-score normalizing each cytokine. This scale-independent composite score showed a similar trend toward higher values in AAA-VoCs, although it did not reach statistical significance (*P* = 0.0626; Fig. S7). These results reveal that AAA-VoCs show a trend toward increased expression for a variety of cytokines tested.

### AAA-VoCs exhibit increased vascular leakage

Endothelial dysfunction is a key feature of AAA, and endothelial barrier promoting genes are downregulated in human aortic AAA tissue (39). We found that in 2D cultures, hiPSC-ECs show decreased junctional integrity after exposure to AV-M (Fig. 5D). To study this further, we performed live cell imaging on day 7 VoCs (Videos S1 and S2). Vascular structures were highlighted using UAE-I, and microvascular lumens were perfused with fluorescein isothiocyanate-conjugated dextran (70 kDa FITC–dextran) (Fig. 5L). In line with our initial findings that microvascular structures are lumenized, we confirmed that both C-VoCs and AAA-VoCs are perfusable. However, when quantifying vascular permeability, we found that AAA-VoCs show an increased vascular leakage over time as compared to C-VoCs (Fig. 5M). These findings show that AAA-VoCs recapitulate a further aspect of AAA *in vitro*, namely endothelial dysfunction, reflected by impaired endothelial barrier integrity.

## Discussion

In this study, we describe the development of a 3D self-organized microvascular model that captures key aspects of VSMC-EC communication by co-culturing primary VSMCs from healthy donors or AAA patients with hiPSC-ECs.

To allow a robust comparison, we used a single, healthy hiPSC-derived EC line in all experiments, resulting in an isogenic microvasculature, supported by either C- or AAA-VSMC lines. We show that VSMCs from both control and AAA donors remain viable after 7 days of VoC culture, respond to ET-1 stimulation, and allow hiPSC-ECs to align in the direction of flow. Importantly, we found that AAA-VSMCs preserve a disease-like phenotype as reflected by an increase in cell number, microvessel surface area, cytokine production, and induction of microvascular permeability.

In our model, VSMCs are distributed throughout the hydrogel and are organized as single cells toward the microvascular structure established herein. This organization resembles the microvasculature *in vivo*, and in particular reflects aspects of the vasa vasorum, where small vessels interact with surrounding smooth muscle cells through localized contact points rather than a continuous mural layer. In contrast, the aortic wall is characterized by a highly organized, multilayered VSMC structure. While our model does not recapitulate this concentric medial architecture and, therefore, represents a simplified system, it is specifically designed to mimic the microvascular niche of the vasa vasorum and to study localized VSMC–EC crosstalk rather than the full aortic wall structure.

Previous studies showed that the average vascular diameter in self-organizing networks depends on ECs, mural cell signaling, hydrogel composition, and the microfluidic platform used (40, 41). Regarding mural cell signaling, we observed several EC–VSMC contact points in both C-VoCs and AAA-VoCs. Contact-dependent EC-VSMC signaling is well established as being essential for vascular maintenance (42), including in the microcirculation (43), and on-chip studies have shown that ECs cultured without supporting mural cells form unstable, poorly organized networks (24). In line with this, the contact points observed in our model likely represent functional EC–VSMC contact points that support vascular network formation after seeding through both direct cell–cell communication and paracrine signaling. Consistent with the similar number of contact sites, we did not detect differences in network complexity between C-VoCs and AAA-VoCs. While we cannot determine whether cytokine expression is localized to these interfaces, the inflammatory phenotype observed is likely driven by a combination of contact-dependent and diffusible signaling within the microenvironment. In the current model, hiPSC-ECs in co-culture with primary VSMCs formed microvascular structures with an average vascular diameter of 54.50 μm (C-VoC) and 65.44 μm (AAA-VoC). It has previously been shown that IL-8 directly induces endothelial cell proliferation (44). Given the increased hiPSC-EC proliferation that we observed in AAA-VoCs at day 7, and the increased IL-8 expression we detected in AAA-VSMCs, it is interesting to speculate that AAA-VSMC-derived paracrine cues drive endothelial expansion, resulting in an increased vascular diameter.

We furthermore quantified EC polarization and compared 2D flow conditions with 3D VoC models. By comparing the 3D system with a defined 2D flow setup with laminar flow, the 2D condition provides a robust technical control for laminar shear stress. A potential limitation is the undefined shear rate in the 3D model. A study using the same microfluidic platform and gravity-driven flow, as employed here, reported a calculated wall shear stress of 0.056–0.14 dynes/cm^2^ with a flow velocity of 0.05–0.13 mm/s and a time of flow duration of 14–18 min (24). In our system, media flow is re-established every 24 h, with a defined spatial fluidic gradient, establishing a right-to-left hydrostatic pressure and flow. Although we did not investigate the shear stress values in our model, we estimate it to be in a comparable range, markedly lower than the 18 dynes/cm^2^ in our 2D ibidi flow system. It was, therefore, contrary to our expectations that flow alignment in the VoC was even more pronounced. The planar cell polarity (PCP) pathway is a non-canonical Wnt pathway that determines PCP in ECs (45). Although strongly activated by LSS (46), the PCP is also active during angiogenesis (47) and was suggested to play a role in vasculogenesis (48). It is likely that the strong polarity observed in our models arises from multiple PCP-activating cues compared to the 2D culture, where shear stress is the only PCP activator. VSMCs may contribute to these cues by providing contact-mediated signals and paracrine factors that regulate endothelial cytoskeletal organization and junctional integrity, processes closely linked to coordinated polarity, including via pathways such as angiopoietin–Tie2 (49). The reduction of PI in branched areas of the VoC, however, indicates that shear stress plays a role in our system, as reduced or disturbed flow, correlates with a reduction of planar polarity (30).

We furthermore found that AAA-VSMCs were higher in number at day 7 compared to C-VSMCs and showed an increased surface area. We did not find an increased number of Ki67-positive nuclei at this timepoint (Fig. S5). We also found no significant differences in AAA-VSMC proliferation or surface area in standard 2D cultures (Fig. S8). These results indicate that the increased cell count and surface area at day 7 are established early after VSMCs are embedded in the hydrogel and co-cultured with hiPSC-ECs. When comparing ET-1-induced calcium transients in C- and AAA-VoCs, we found comparable transients for VSMCs in both models. In AAA, VSMCs shift toward a proliferative, less contractile phenotype (50), though the extent of contractile loss remains unclear. A recent *in vitro* study of AAA patient-derived VSMCs reported reduced contraction in only 23% of cases, suggesting this occurs but is relatively infrequent (25).

Finally, we evaluated AAA-associated VSMC and EC dysfunction *in vitro*. *In vivo* studies have shown that VSMCs in AAA produce elevated levels of pro-inflammatory cytokines (15, 16). Furthermore, previous studies show that endothelial barrier function is compromised in AAA and correlates with disease severity, whereas improving the barrier attenuates early AAA development (39, 51). AAA-VoCs showed a trend toward higher cytokine production than C-VoCs. Because these increases were modest in the absence of immune cells, we used a composite score to capture coordinated cytokine shifts while recognizing the limitations of summing individually small effects. We found CCL2 showing the most pronounced differential expression for AAA-VoCs. CCL2 is upregulated in a rodent model of AAA (52). Moreover, a recent study performing RNAseq shows that CCL2 is differentially expressed in AAA (53). We also found that IL-8 is upregulated in our 3D model and in 2D AAA-VSMCs, and an upregulation of IL-8 in human AAA biopsies was reported previously (54).

The relatively low expression of IL-6 in 2D hiPSC-ECs, as well as AAA-VoCs, was unexpected due to previous reports of IL-6 upregulation in rodent AAA models and patient samples (55, 56). Given the multicellular role of AAA, ECs may contribute only minimally to overall IL-6 levels. It is also known that VSMCs upregulate IL-6 in response to fibrin degradation products (57, 58), which could be a confounding factor, especially after 7 days of culture in fibrin hydrogel. Finally, we found that the overall PAI-1 secretion was specifically high in both C-VoCs and AAA-VoCs. Because PAI-1 protects VSMCs from apoptosis, we speculate that these elevated levels reflect the relatively artificial VSMC environment in our model (59). Finally, the modest differences in cytokine levels observed between C-VoCs and AAA-VoCs may be related to the minimal shear stress present in our model, as samples were collected after 24 h under low-shear levels in the VoC model. Given the vasoprotective role of physiological shear stress, its presence could reduce baseline cytokine expression (e.g. in C-VoCs), thereby potentially enhancing the relative differences between C-VoCs and AAA-VoCs and allowing VSMC-specific effects to be more clearly resolved. Altogether, we find in our model a cytokine expression profile for AAA-VoC that, to some extent, recapitulates findings from prior human patient studies; however, it is observed as a generalized trend, rather than statistically significant for individual cytokines.

Finally, we observed an increase in vascular leakage in AAA-VoCs. This may relate to the increased expression of IL-8, which induces proliferation in endothelial cells (44), correlating with impaired barrier stability (60). Together, these findings suggest that increased proliferation, cytokine burden, and junctional impairment underlay the observed increase in vascular leakage in AAA-VoCs. As previous studies showed that improving endothelial barrier integrity can slow AAA progression, these findings suggest that our platform may offer a promising avenue for screening compounds that target endothelial stabilization.

## Supplementary materials









## Declaration of interest

The authors declare that there is no conflict of interest that could be perceived as prejudicing the impartiality of the work reported.

## Funding

PCH was supported by the Amsterdam UMC. This work was furthermore supported by the Netherlands Organ-on-Chip Initiative (024.003.001) funded by the Ministry of Education, Culture and Science of the government of the Netherlands. KKY was supported by the Senior Clinical Scientist grant (Dekkerbeurs 2019T065).
