# Supplementary material for: Modeling smooth muscle cell–endothelial cell crosstalk in abdominal aortic aneurysms using 3D microvessels on-chip
Source: Vasc Biol. 2026 May 7;8(1):VB260005. doi: 10.1530/VB-26-0005 (PMC13178432; doi:10.1530/VB-26-0005)
Supplement: Supplementary file 1 [file supplementary_figures.pdf]

Supplementary Figure S1

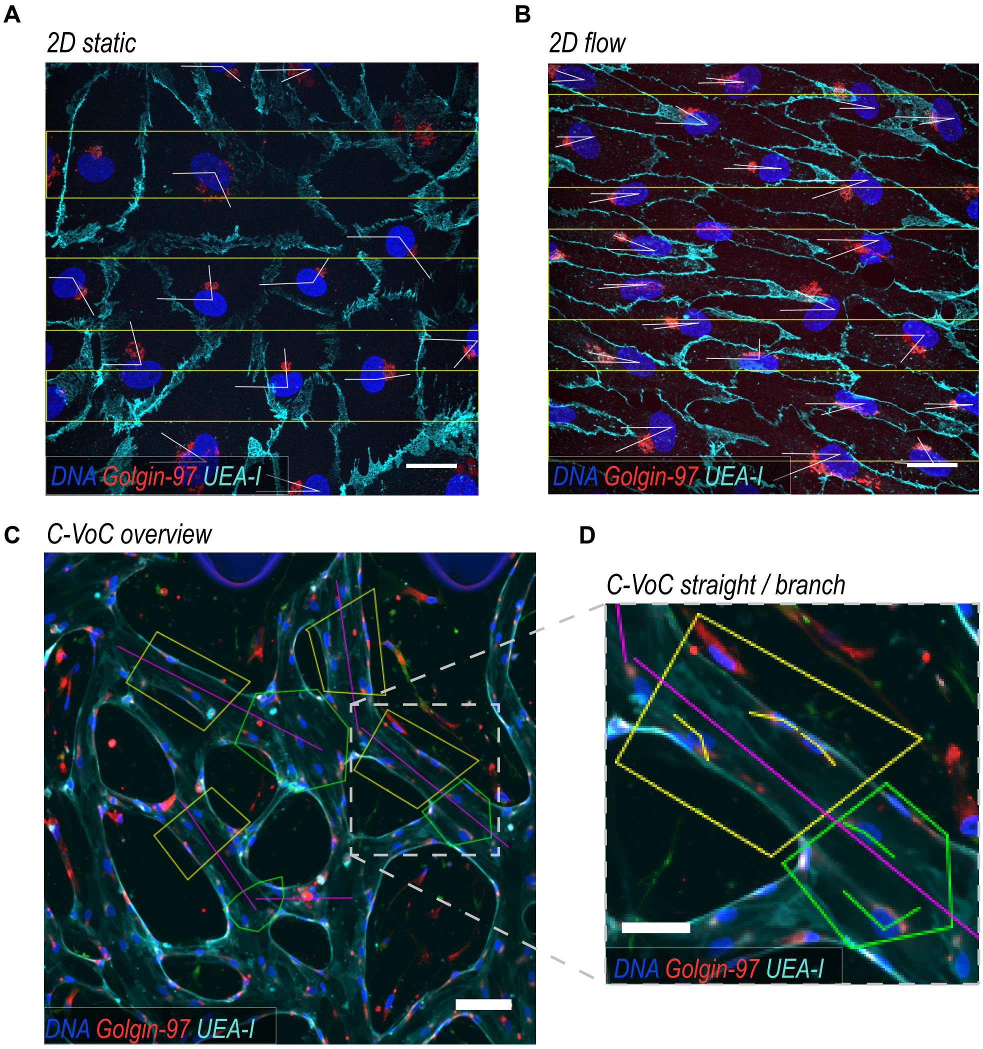

Figure S1: Golgi-to-nuclei angle determination gridlines and microvascular branch labeling
(A) Representative confocal images of static hiPSC-ECs in 2D for Golgi-to-nuclei orientation analysis including parallel lines (yellow) as reference for golgi-to-nuclei angle determination (white angle indications) (gray: DAPI; red: Golgin – 97, cyan: VE-Cadherin). Scale bar: 25 µm. (B) Representative confocal images of hiPSC-ECs after 72h of flow in 2D for Golgi-to-nuclei orientation analysis including parallel lines (yellow) as reference for golgi-to-nuclei angle determination (white angle indications) (gray: DAPI, red: Golgin – 97, cyan: VE-Cadherin). Scale bar: 25 µm. (C) Representative confocal image of VoC for Golgi-to-nuclei orientation analysis including straight (yellow) and branched (green) labelled areas and reference line (magenta) parallel to vessel wall. (gray: DAPI, red: Golgin – 97, cyan: UEA-I). Scale bar: 100 µm. (D) Close up image (gray dashed indicated area indicated in (C)) of representative straight (yellow) and branched (green) labelled areas in VoC. Magenta line indicates reference line parallel to vessel wall. Golgi-to-nuclei angle determination parallel to reference line in the straight labelled area indicated in yellow angle, in branched areas indicated with a green angle. (gray: DAPI, red: Golgin – 97, cyan: UEA-I). Scale bars: 50 µm.

Supplementary Figure S2

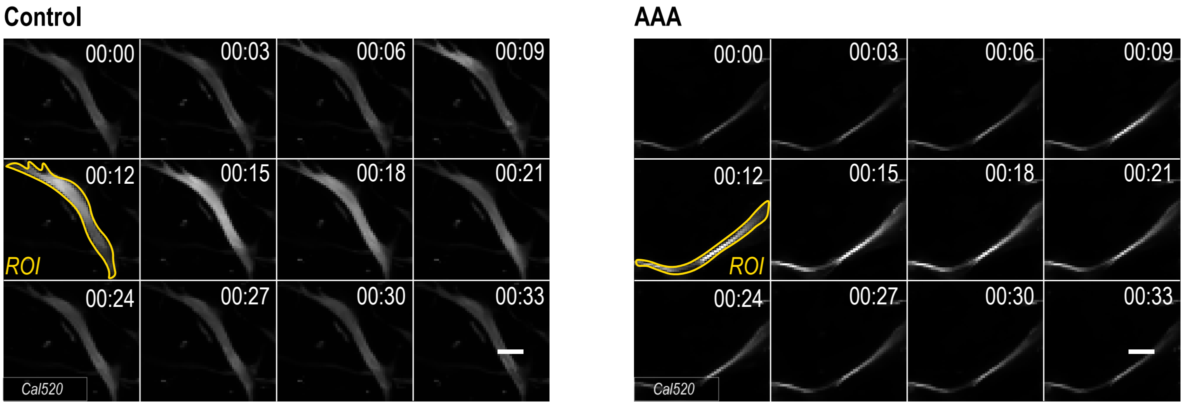

Figure S2: Ca2+ transient imaging and ROI determination
Representative image for ROI detection of C-VSMC (left panel) and AAA-VoC (right panel) in VoC 5 minutes after ET-1 stimulation over the time course of 33 seconds. Representative ROI in yellow outline. (Time stamp: MM:SS, gray: Cal520). Scale bars: 25 µm.

Supplementary Figure S3

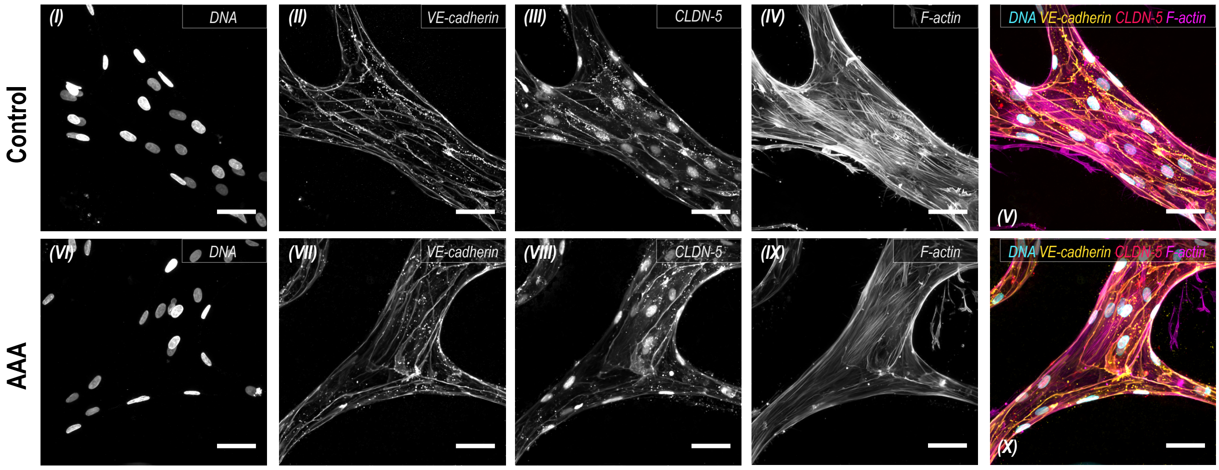

Figure S3: Tight and Adherens junction expression of hiPSC-ECs in Vessel-on-Chip
Representative confocal images of microvascular networks, upper panel in C-VoC, lower panel in AAA-VoC. (I + VI) cell nuclei, (II + VII) VE-Cadherin, (III + VIII) CLDN-5 and (IV + IX) F-actin. (V) Composite images for C-VoC and (X) AAA-VoC (blue: DAPI, yellow: VE-Cadherin, red: CLDN-5, magenta: F-actin). Scale bars: 50 µm.

Supplementary Figure S4

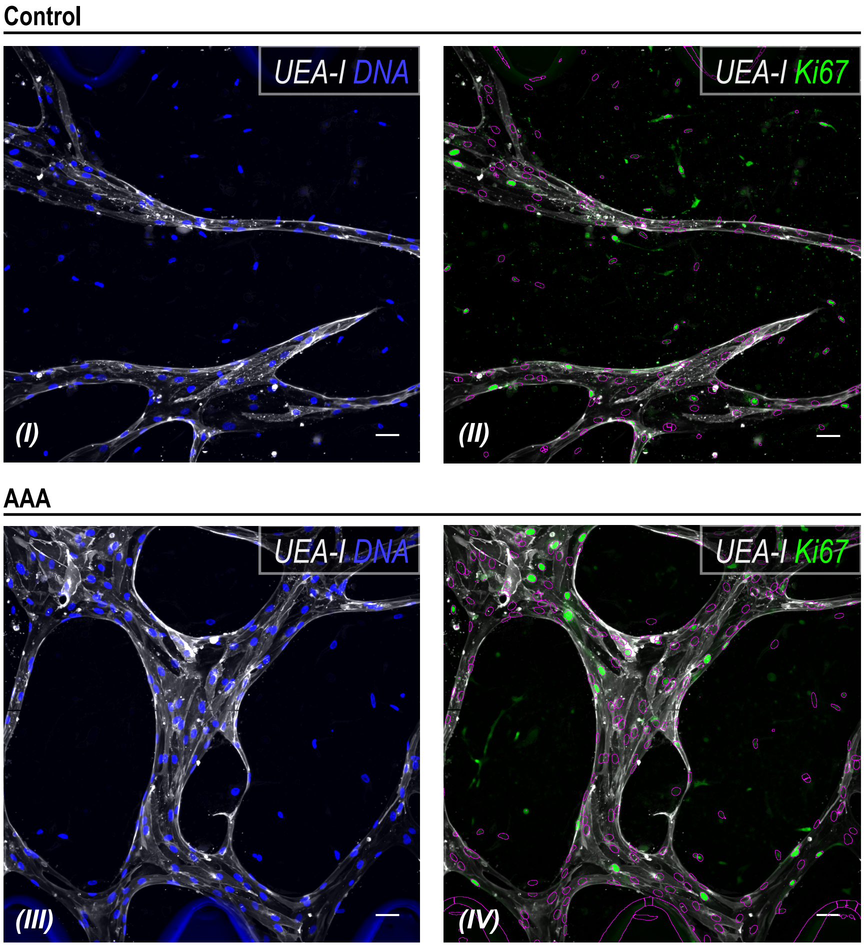

Figure S4: Ki67 analysis on VoC platform
(I–IV) Representative confocal images of VoC for Ki67 analysis. (I) C-VoC showing nuclei for ROI determination (blue: DAPI, gray: UEA-I). (II) C-VoC with ROI boundaries (pink lines, outlining cell nuclei) defined by thresholding the DNA stain in (I) (green: Ki67, gray: UEA-I). (III) AAA-VoC showing nuclei for ROI determination (blue: DAPI, gray: UEA-I). (IV) AAA-VoC with ROI boundaries (pink lines outlining cell nuclei) defined by thresholding the DNA stain in (III) (green: Ki67, gray: UEA-I). Scale bars: 50 µm.

Supplementary Figure S5

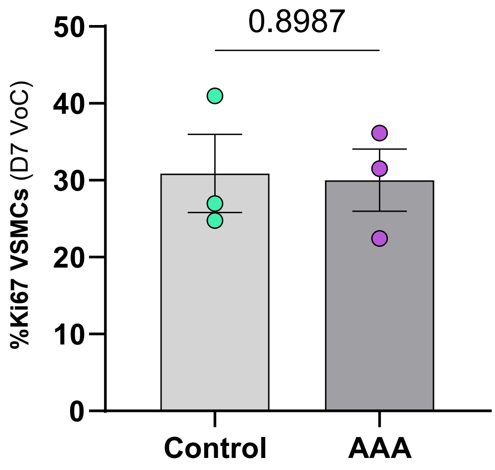

Figure S5: Fraction of Ki67 positive nuclei in VSMCs
Quantification of fraction of Ki67 positive VSMC nuclei in VoC culture at day 7 in %. Data are shown as mean ± SEM. Data points represent 3 independent experiments on 3 (control) and 3 (AAA) independent VSMC lines; 3–6 VoC channels per experiment were imaged and data averaged to yield one value per cell line. Data passed Shapiro–Wilk normality test, groups compared using unpaired two-tailed t-test. *p< 0.05.

Supplementary Figure S6

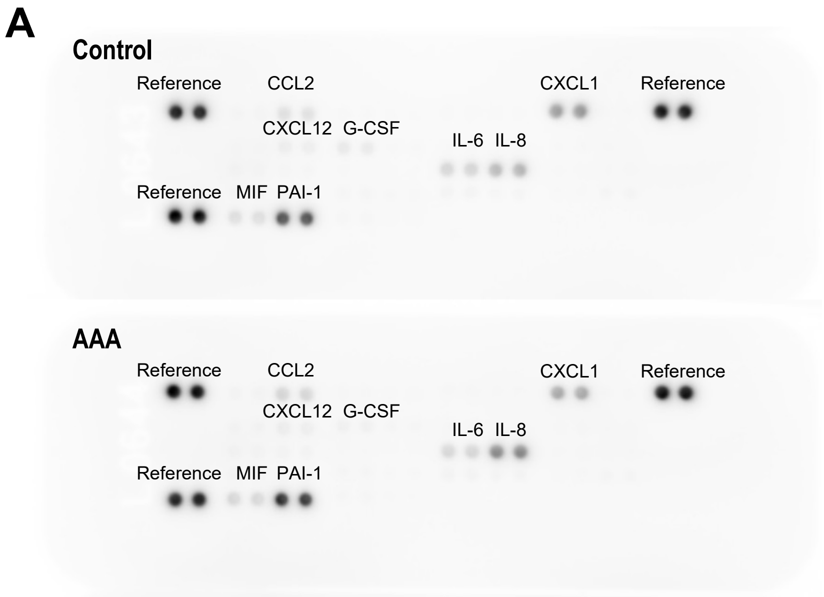

Figure S6: Human cytokine array detection membrane
(A) Representative human cytokine array detection membrane after incubation with C-VoC supernatant (top panel) or AAA-VoC (bottom panel).

Supplementary Figure S7

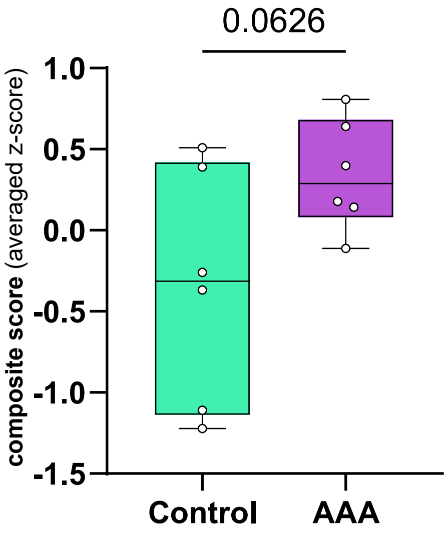

Figure S7: Z-scored composite score of cytokine expression
Composite score of cytokine expression levels after log-transformation and z-score normalization across all samples within each group. Composite score values are averaged per sample. Data are shown as box plots: median (line), interquartile range (box), and minimum to maximum values. Each point represents an individual sample (n = 3 independent VSMC lines, 2 technical replicates each).

Supplementary Figure S8

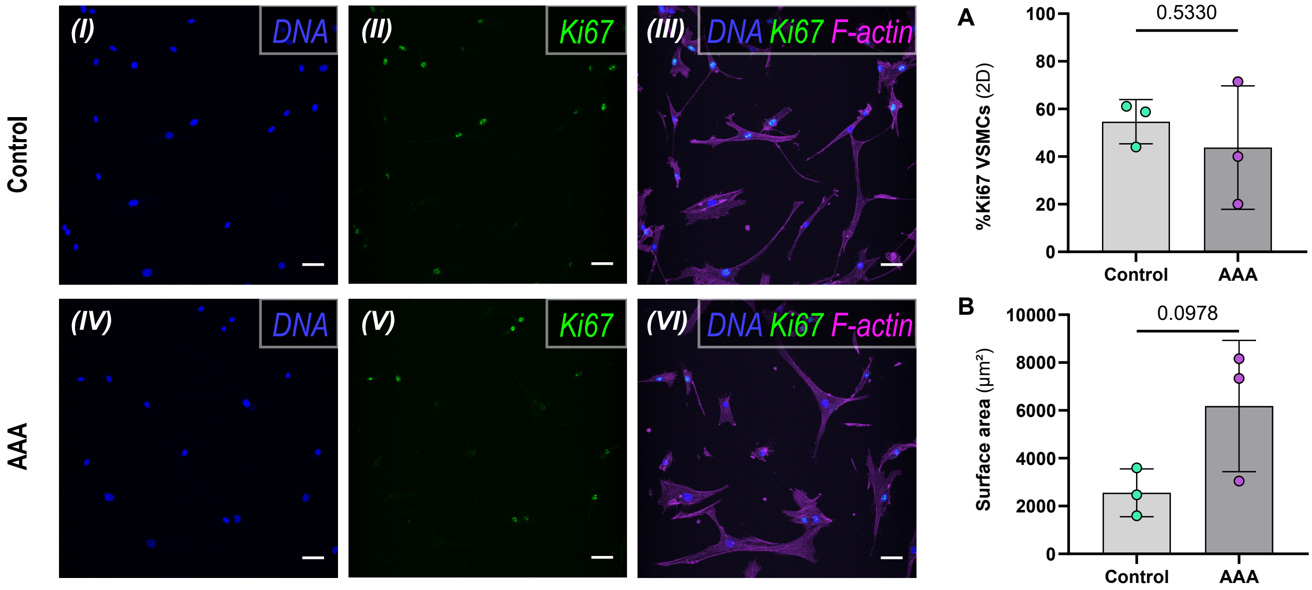

Figure S8: VSMC proliferation and surface area in 2D
(I–IV) Representative confocal images C-VSMCs (I-III) and AAA-VSMCs (IV-VI) in 2D culture. (I + VI) cell nuclei, (II + VII) Ki67, (III + VIII) Composite (blue: DNA, green: Ki67. Magenta: F-actin). (A) Quantification of Ki67% positive cell nuclei for C-VSMCs and AAA-VSMCs. (B) Quantification of surface area in µm² for C-VSMCs and AAA-VSMCs in 2D, ROI selection based on F-actin staining on 2D maximum intensity projection images. Data are shown as mean ± SD. Data points represent 3 independent experiments on 3 (control) and 3 (AAA) independent VSMC lines, 1 image per cell line. Data passed Shapiro–Wilk normality test, groups compared using unpaired two-tailed t-test, significance level $p < 0.05$. Scale bars: 50 µm.
