# Supplementary material for: Modeling smooth muscle cell–endothelial cell crosstalk in abdominal aortic aneurysms using 3D microvessels on-chip
Source: Vasc Biol. 2026 May 7;8(1):VB260005. doi: 10.1530/VB-26-0005 (PMC13178432; doi:10.1530/VB-26-0005)
Supplement: Supplementary file 2 [file supplementary_tables.pdf]

Tables:

Table S1: Included cell lines

Endothelial Cells										
**Donor**	**Type**	**Line Name**	**Sex**	**Age**	**Smoking**	**Diabetes**	**Genetic diagnosis**	**Hypertension**	**Hypercholesterolemia**	**Renal dysfunction**
Control	hiPSC-EC	SCVI111	M	26	NA	NA	NA	NA	NA	NA
**Vascular Smooth Muscle Cells**										
**Donor**	**Type**	**Line Name**	**Sex**	**Age**	**Smoking**	**Diabetes**	**Genetic diagnosis**	**Hypertension**	**Hypercholesterolemia**	**Renal dysfunction**
Control	primary VSMC	7-10-2019	M	53	NA	NA	NA	NA	NA	NA
Control	primary VSMC	23-1-2024	M	68	NA	NA	NA	NA	NA	NA
Control	primary VSMC	28-3-2024	M	40	NA	NA	NA	NA	NA	NA
Control	primary VSMC	19-4-2024	F	61	NA	NA	NA	NA	NA	NA
Control	primary VSMC	15-4-2022	F	76	NA	NA	NA	NA	NA	NA
Control	primary VSMC	11-11-2019	F	35	NA	NA	NA	NA	NA	NA
Control	primary VSMC	19-8-2021	F	67	NA	NA	NA	NA	NA	NA
Control	primary VSMC	27-3-2025	F	59	NA	NA	NA	NA	NA	NA
Control	primary VSMC	18-2-2020	F	47	NA	NA	NA	NA	NA	NA
Control	primary VSMC	1-11-2021	M	53	NA	NA	NA	NA	NA	NA
Control	primary VSMC	29-11-2023	F	42	NA	NA	NA	NA	NA	NA
Control	primary VSMC	16-11-2023	F	60	NA	NA	NA	NA	NA	NA
Control	primary VSMC	4-5-2025	F	66	NA	NA	NA	NA	NA	NA
Control	primary VSMC	3-6-2024	F	60	NA	NA	NA	NA	NA	NA
Control	primary VSMC	16-2-2024	M	65	NA	NA	NA	NA	NA	NA
Control	primary VSMC	24-5-2024	F	66	NA	NA	NA	NA	NA	NA
Control	primary VSMC	29-8-2025	NA	under 18	NA	NA	NA	NA	NA	NA
Control	primary VSMC	22-6-2025	M	73	NA	NA	NA	NA	NA	NA
Control	primary VSMC	26-6-2025	F	27	NA	NA	NA	NA	NA	NA
AAA	primary VSMC	28-11-2024	F	67	Yes	No	No	No	No	No
AAA	primary VSMC	23-4-2024	F	57	Yes	No	No	Yes	No	No
AAA	primary VSMC	16-4-2024	M	78	Stopped	No	No	No	No	No
AAA	primary VSMC	25-11-2024	M	66	No	No	No	Yes	Yes	Yes
AAA	primary VSMC	19-12-2016	M	76	Yes	Yes	No	Yes	Yes	No
AAA	primary VSMC	21-8-2019	F	71	Stopped	No	No	Yes	Yes	No
AAA	primary VSMC	29-5-2017	M	76	Stopped	No	No	No	No	No
AAA	primary VSMC	21-7-2020	M	85	Stopped	No	No	Yes	No	Yes
AAA	primary VSMC	1-3-2017	M	60	Stopped	No	No	Yes	No	No
AAA	primary VSMC	25-8-2021	M	53	Yes	Yes	No	No	No	Yes
AAA	primary VSMC	16-1-2024	M	70	Stopped	No	No	No	Yes	Yes
AAA	primary VSMC	12-5-2025	M	54	Stopped	No	No	No	No	No
AAA	primary VSMC	10-2-2017	F	83	Yes	No	No	Yes	No	No
AAA	primary VSMC	18-12-2024	M	65	Stopped	No	No	Yes	No	No

AAA	primary VSMC	1-9-2025	V	73	Stopped	No	No	Yes	No	No
AAA	primary VSMC	9-5-2023	M	57	Stopped	No	No	Yes	No	No
AAA	primary VSMC	11-7-2023	M	52	No	No	No	No	No	No

Table S2: Cell lines per experiment

Figure	primary VSMC	
	Control	AAA
2B-E	7-10-2019<br>18-2-2020<br>23-1-2024<br>28-3-2024<br>15-4-2022	21-7-2020<br>1-3-2017<br>16-1-2024<br>16-1-2024<br>16-4-2024
2F, S7	19-4-2024<br>15-4-2022<br>28-3-2024	23-4-2024<br>16-4-2024<br>16-1-2024
3D-G	15-4-2022<br>29-11-2023<br>16-11-2023	16-4-2024<br>25-11-2024<br>28-11-2024
4C-F	19-4-2024<br>15-4-2022<br>15-4-2022<br>29-11-2023	23-4-2024<br>16-4-2024<br>16-4-2024<br>25-11-2024
5A-F	29-8-25<br>22-6-25<br>26-6-25	1-9-25<br>9-5-23<br>11-7-23
5H-F, S5, S6	11-11-2019<br>19-8-2021<br>27-3-2025	19-12-2016<br>21-8-2019<br>29-5-2017
5L-M	18-2-2020<br>1-11-2021<br>29-11-2023<br>16-11-2023	21-7-2020<br>1-3-2017<br>25-8-2021<br>16-1-2024<br>16-4-2024
S8 (2D culture only)	3-6-2024<br>16-2-2024<br>24-5-2024	10-2-2017<br>18-12-2024<br>28-11-2024

Table S3: Antibody list

Antibody/Probe	Label	Supplier	Cat. No.	Fixation	Dilution	Usage
DAPI	DAPI	Thermo Fisher Scientific	62248	4% PFA	1:600	ICC
UEA I	DyLight® 649	Vector labs	DL-1068-1	NA	1:500	Live Cell Imaging
UEA I	DyLight® 649	Vector labs	DL-1068-1	4% PFA	1:500	ICC
Phalloidin	Acti-stain™ 670	Cytoskeleton, Inc.	PHDN1	4% PFA	1:600	ICC
VE-cadherin	NA	Cell Signalling	D87F2	4% PFA	1:500	ICC
CD31 (PECAM-1)	NA	Abcam	ab28364	4% PFA	1:500	ICC
Claudin-5	NA	Invitrogen	34-1600	4% PFA	1:500	ICC
Golgin-97	NA	Invitrogen	14-976-782	4% PFA	1:500	ICC
Transgelin (SM22)	NA	Abcam	ab155272/ab14106	4% PFA	1:500	ICC
α smooth muscle actin	NA	Abcam	ab21027	4% PFA	1:500	ICC
Ki67	NA	Sigma-Aldrich	AB9260	4% PFA	1:500	ICC
ICAM-1/CD54 Polyclonal antibody	NA	Proteintech	10831-1-AP	4% PFA	1:500	ICC
VCAM-1/CD106 Monoclonal antibody	NA	Proteintech	66294-1-Ig	4% PFA	1:500	ICC
NA	Alexa Fluor™ 488	Invitrogen	A21206	4% PFA	1:400	ICC
NA	Alexa Fluor™ 555	Invitrogen	A31570	4% PFA	1:400	ICC

Table S4: Primer sequences for quantitative PCR

Name	Sequence (5' - 3')
IL-8_fw	CTGGCCGTGGCTCTCTTG
IL-8_rv	CTTGGCAAAACTGCACCTTCA
IL-6_fw	CCCACACAGACAGCCACTCA
IL-6_rv	CCGTCGAGGATGTACCGAAT
CCL2_fw	GCTCAGCCAGATGCAATCAA
CCL2_rv	TTCTTTGGGACACTTGCTGC
PAI-1_fw	CACAAATCAGACGGCAGCACT
PAI-1_rv	CATCGGGCGTGGTGAACTC
GAPDH_fw	AGCCACATCGCTCAGACAC
GAPH_rv	GCCCAATACGACCAAATCC
